# Eco-Engineered Biopolymer–Clay Composite for Phosphate IonRemoval: Synergistic Insights from Statistical and AI Modeling

**DOI:** 10.3390/polym17131805

**Published:** 2025-06-28

**Authors:** Rachid Aziam, Daniela Simina Stefan, Safa Nouaa, Mohamed Chiban, Mircea Stefan

**Affiliations:** 1 Department of Analytical Chemistry and Environmental Engineering, Faculty of Chemical Engineering and Biotechnologies, National University of Science and Technology Politehnica of Bucharest, 1−7 Polizu Street, 011061 Bucharest, Romania; rachid.aziam@edu.uiz.ac.ma; 2 Laboratory of Applied Chemistry and Environment, Department of Chemistry, Faculty of Science, Ibnou Zohr University, Agadir BP 8106, Morocco; safa.nouaa@edu.uiz.ac.ma; 3 Pharmacy Faculty, University Titu Maiorescu, No. 22 Dâmbovnicului Street, District 4, 040441 Bucharest, Romania; mircea.stefan@prof.utm.ro

**Keywords:** bio-composite beads, alginate, iota-carrageenan, natural clay, phosphate ions, ANN, RSM

## Abstract

This research aims to synthesize a novel hydrogel bio-composite based on natural clay, sodium alginate (Na-AL), and iota-carrageenan as adsorbents to remove phosphate ions from aqueous solutions. The adsorbents were characterized by a variety of techniques, such as Fourier-transform infrared (FTIR) spectroscopy, scanning electron microscopy coupled with energy dispersive X-rays (SEM-EDX), and the determination of point zero charge (PZC). This research investigated how the adsorption process is influenced by parameters such as adsorbent dose, contact time, solution pH, and temperature. In this study, we used four isotherms and four kinetic models to investigate phosphate ion removal on the prepared bio-composite. The results showed that the second-order kinetic (PSO) model is the best model for describing the adsorption process. The findings demonstrate that the R^2^ values are highly significant in both the Langmuir and Freundlich models (very close to 1). This suggests that Langmuir and Freundlich models, with a diversity of adsorption sites, promote the adsorption of phosphate ions. The maximum adsorbed amounts of phosphate ions by the bio-composite used were 140.84 mg/g for H_2_PO_4_^−^ ions and 105.26 mg/g for HPO_4_^2−^ ions from the batch system. The positive ∆H° confirms the endothermic and physical nature of adsorption, in agreement with experimental results. Negative ∆G° values indicate spontaneity, while the positive ∆S° reflects increased disorder at the solid–liquid interface during phosphate uptake. The main parameters, including adsorbent dosage (mg), contact time (min), and initial concentration (mg/L), were tuned using the Box–Behnken design of the response surface methodology (BBD-RSM) to achieve the optimum conditions. The reliability of the constructed models is demonstrated by their high correlation coefficients (R^2^). An R^2^ value of 0.9714 suggests that the model explains 97.14% of the variability in adsorption efficiency (%), which reflects its strong predictive capability and reliability. Finally, the adsorption behavior of phosphate ions on the prepared bio-composite beads was analyzed using an artificial neural network (ANN) to predict the process efficiency. The ANN model accurately predicted the adsorption of phosphate ions onto the bio-composite, with a strong correlation (R^2^ = 0.974) between the predicted and experimental results.

## 1. Introduction

Today, eliminating phosphate ions from industrial effluents is a major challenge for environmental protection and sustainable water management. Phosphorus, being a nonrenewable resource, faces challenges due to its uneven global distribution and rapid exploitation, which significantly threaten human survival and agricultural production [1,2]. The main factor in the eutrophication of surface waters is the presence of excess phosphate ions, which occur even at a soluble reactive P concentration of 0.02 mg/L [3,4].

Phosphorus is strongly chemically bound in aquatic environments, mainly by multivalent metals, giving rise to complexes and insoluble salts.

A range of techniques have been utilized for phosphate ion removal from wastewater, such as membrane filtration, photodegradation, bioremediation, ultrafiltration, and adsorption [5,6,7,8]. Adsorption has been proven to be an effective treatment technique due to its low capital cost, simple concept, ease of use, and insensitivity to chemical contaminants. However, the high cost of some adsorbents and the quantity of wastewater involved limit their application. Adsorption using polymer-based bio-composites as efficient, affordable, and environmentally friendly adsorbents is the most appropriate and effective method for removing phosphate ions [9,10].

Natural clays are highly effective and selective in removing phosphate ions from water. However, their large surface area leads to rapid agglomeration, making their extraction from aqueous solutions challenging [11,12]. Bentonite (Bent), a widely available clay mineral, holds significant importance in environmental engineering due to its affordability, nanoscale layer spacing, high cation exchange capacity, and extensive surface area. Its permanent negative charge arises from isomorphous substitutions, where Al^3+^ replaces Si^4+^ in the tetrahedral layer and Mg^2+^ replaces Al^3+^ in the octahedral layer [13,14].

Alginate and iota-carrageenan were used as bio-polymeric supports for natural clay in the present work. Alginate is an eco-friendly, biodegradable, and cost-effective natural polysaccharide known for its non-toxic properties [5,11,12,15]. Carrageenan is composed of a repeating disaccharide structure, alternating between D-galactose and 3,6-anhydro-galactose. It comes in three primary forms: kappa (kCR), iota (iCR), and lambda (lCR), distinguished by the number of sulfate groups per disaccharide unit, with one in kappa, two in iota, and three in lambda [16,17,18].

The use of iota-type carrageenan (iCR) in the synthesis of the bio-composite prepared in this study is attributed to its excellent gelling properties. As a result, the most commonly utilized carrageenan types in the industry are kappa-type (kCR) and iota-type (iCR) [16].

Aziam et al. (2023) and Zhao et al. (2023) have highlighted that coordination polymers are particularly effective at detecting trace amounts of environmental toxins. Their desirable properties, such as simple synthesis, quick responsiveness, and high sensitivity, contribute to their suitability for this application [12,19]. Numerous studies have confirmed the efficiency of alginate-based bio-composites in water treatment, particularly for eliminating both organic and inorganic contaminants. Bio-composite beads exhibit superior removal efficiency compared to many traditional adsorbents, and they have been tested as a new nanomaterial that can be used for water purification [20,21,22].

The response surface methodology (RSM) is an excellent strategy for reducing the number of experimental attempts and furnishing more informative datasets. The RSM is a statistical tool used to model and optimize complex processes. It enhances the precision of predicted responses while reducing the number of required experimental trials [23].

Artificial neural networks (ANNs) are effective tools for modeling complex functional interactions such as adsorption processes. Their advantages, such as strong predictive accuracy, adaptability, and the ability to handle non-linear, multivariable systems, make them well-suited for adsorption studies. These characteristics have confirmed their reliability in simulating and analyzing adsorption behavior [24].

This research aims to synthesize a novel hydrogel bio-composite based on natural clay, sodium alginate (Na-AL), and kappa-carrageenan as adsorbents to remove phosphate ions from aqueous solutions through response surface methodology and artificial neural network modeling. The adsorbents were characterized by Fourier-transform infrared (FTIR) spectroscopy, scanning electron microscopy coupled with energy dispersive X-rays (SEM-EDX), and the determination of point zero charge (PZC).

This study investigated how the adsorption process is influenced by factors such as adsorbent dose, contact time, solution pH, and temperature. The kinetics, isotherms, and thermodynamics of the process were studied. The main parameters, including adsorbent dosage (mg), contact time (min), and initial concentration (mg/L), were tuned using the Box–Behnken design of the response surface methodology (BBD-RSM) to achieve the optimum conditions. Finally, the phosphate ion adsorption behavior of the prepared bio-composite beads was analyzed using an artificial neural network (ANN) to predict the process efficiency. This study offers a novel research approach to exploring the adsorption mechanism of bio-composite surfaces.

## 2. Materials and Methods

### 2.1. Synthesis of Bio-Composite Beads

**Figure 1:**
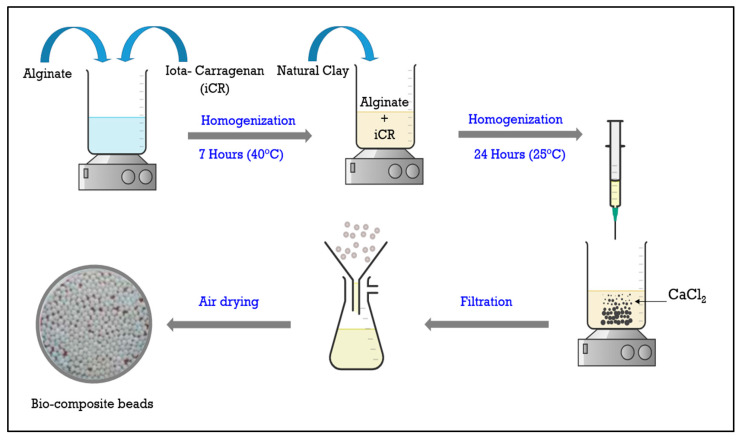
Synthesis of bio-composite beads.

The bio-composite beads used in this study were developed using the extrusion synthesis method, as shown in the diagram below (Figure 1).

In order to prepare the bio-composite beads, 1 g of alginate and 1 g of iota-carrageenan were continuously stirred with double-distilled water in a 100 mL Erlenmeyer flask at 40 °C for 7 h. The solutions were agitated at 500 rpm to ensure the complete dispersion of the alginate and carrageenan. Afterward, 2 g of natural bentonite clay was added to the mixture, which was then gently stirred at room temperature. The resulting solution, containing alginate, carrageenan, and clay, was transferred into a syringe for bead formation. The syringe was positioned upright over a 0.1 M calcium chloride (CaCl_2_) solution for gelation. The mixture was introduced drop by drop into the gel bath, causing instant gelation as polymer chains accumulated around Ca^2+^ cations. The beads, along with the calcium chloride solution, were left undisturbed for a sufficient maturation period to allow complete gelation. Once matured, the beads were collected by filtration, thoroughly rinsed with distilled water, and then directly used in their hydrogel form [11,12].

### 2.2. Adsorption Experiments

Adsorption experiments were carried out in batch mode at room temperature, except for tests assessing the influence of temperature. This approach was chosen for its practicality and effectiveness. A measured amount of bio-composite beads was placed in sealed 100 mL Erlenmeyer flasks containing 50 mL of a phosphate ion solution at a specific concentration and pH. To maintain uniform mixing, the stirring speed remained constant throughout the process.

Following variations in contact time (t), the solutions were centrifugated at 5000 rpm for 10 min. The supernatant was subsequently filtered using a 0.45 μm membrane before undergoing analysis. The residual concentration of phosphate ions in the solution was assessed via UV–visible spectrophotometry, with phosphate ions (H_2_PO_4_^−^ and HPO_4_^2−^) exhibiting a peak absorption wavelength of 700 nm. The amount of phosphate ions removed (Cr) was determined by calculating the difference between the initial concentration (C_0_) and the concentration recorded at various time intervals. The initial pH values were measured at 7.27 for the HPO_4_^2−^ solution and 5.09 for the H_2_PO_4_^−^ solution.

The quantity of adsorbate removed per gram of adsorbent (*q_t_*, mg/g) at a given time *t* was determined using the following equation:

(1) $$q_{t}\left(mg\cdot g^{-1}\right)=\frac{\left(C_{0}-C_{t}\right)}{m}\cdot V$$

The removal percentage of phosphate ions was calculated by:

(2) $$\%\;R=\frac{\left(C_{0}-C_{t}\right)}{C_{0}}\%$$

### 2.3. Adsorption Kinetics and Isotherms

In general, an increase in contact time leads to a rise in the phosphate ion removal rate, but only up to a certain threshold. As phosphate ions accumulate on the available adsorption sites of the adsorbent material, any further extension of contact time does not enhance adsorption efficiency. The influence of contact time plays a crucial role in establishing equilibrium between the adsorbent and adsorbate. Adsorption kinetics help to quantify the amount of the pollutant absorbed over time, illustrating how the adsorption capacity evolves throughout the process. The removal of pollutants from an aqueous solution using an adsorbent can be understood through kinetic modeling, which helps analyze the mechanisms that govern the adsorption rate. These mechanisms may include chemical reactions, diffusion processes, and mass transfer phenomena [5,12,25,26,27].

The various forms of kinetic models, such as pseudo-first-order (PFO), pseudo-second-order (PSO), intra-particle diffusion (IPD), and Elovich models, are illustrated in Table 1.

The parameter *q_t_* (mg/g) refers to the amount of H_2_PO_4_^−^ and HPO_4_^2−^ ions removed at a specific time t. *C*_0_ (mg/L) is the initial concentration of phosphate ions in the solution, while *C_t_* (mg/L) indicates their concentration at each time t. V (L) is the volume of the polluted solution.

Adsorption isotherms give an idea of the adsorption mechanism between phosphate ions and the surface of bio-composite beads. Adsorption isotherms are essential for understanding and optimizing the efficiency of the adsorption process. The analysis of isotherm data through fitting them to various isotherm models is a crucial step in determining a suitable model for design purposes. The equations of non-linear and linear forms for four models—Langmuir, Freundlich, Temkin, and Dubinin–Radushkevich—are given in Table 2 [12,25,28,29,30,31,32].

In the given context, *q_e_* (mg·g^−1^) indicates the amount adsorbed at the equilibrium concentration, *q_L_* (mg·g^−1^) signifies the Langmuir constant denoting the maximum monolayer capacity, and *K_L_* (L·mg^−1^) is the Langmuir constant associated with the energy of adsorption. Additionally, *q_e_* (mg·g^−1^) denotes the amount of phosphate ions adsorbed per unit weight of the adsorbent, *C_e_* (mg·L^−1^) is the equilibrium concentration of the phosphate solution, *K_F_* (mg·g^−1^) is the Freundlich constant, which is a comparative measure of the adsorption capacity of the adsorbent, and n is an empirical constant linked to the heterogeneity of the adsorbent surface. The parameter n also provides insights into the nature of the adsorption process, T represents the absolute temperature in Kelvin, and R is the universal gas constant (8.314 J·K^−1^·mol^−1^). The parameter *b_T_* (J·mol^−1^) relates to the Temkin isotherm constant associated with the heat of adsorption, while *K_T_* (L·mg^−1^) represents the equilibrium binding constant related to the maximum binding energy. *q_m_* (mg·g^−1^) signifies the theoretical saturation capacity, and ε is the Polanyi potential.

### 2.4. Experimental Design Using the Box–Behnken Design

Response surface methodology (RSM) finds extensive applications in evaluating sensitivity functions and establishing optimal correlations among variables through predictive equations. These equations consider that preferred responses may be influenced by the interactions of multiple independent factors [23]. In the current investigation, we employed the Box–Behnken design of response surface methodology (BBD-RSM) through Design Expert 12 software. BBD-RSM is an analytical design used to model experiments and solve complex optimization problems in order to obtain maximum information and the highest possible accuracy of answers calculated by the model using the minimum number of experiments [33]. Three levels (−1, 0, and 1) were utilized to optimize various parameters affecting the removal percentage of phosphate ions. The specific levels for each parameter in the BBD are outlined in Table 3, where A denotes the adsorbent dose (mg), B is the contact time (min), and C is the concentration of phosphate ions (mg/L).

The adsorption efficiency (%) depends on various functional parameters, such as the adsorbent dose (mg) (A), contact time (B), and initial concentration (mg/L) (C). This process was optimized using the Box–Behnken design and response surface methodology, which involved 17 experiments.

A second-order polynomial model that links the response Y to the chosen factors A, B, and C was used (Equation (3)).

(3) $$Y\left(\%\right)=\beta_{0}+\Sigma \;\beta_{i}x_{i}+\Sigma \;\beta_{ij}x_{i}x_{j}+\Sigma \;\beta_{ii}{x_{i}}^{2}+\varepsilon$$

*Y* represents the predicted response, which is the key variable of interest for the experiment and is determined with a certain level of precision. $\beta_{0}$ denotes the constant coefficient or the mean value of the response, while $\beta_{i}$, $\beta_{ij}$, and $\beta_{ii\;}$ correspond to the coefficients of the mathematical model. These coefficients are initially unknown and must be derived from experimental data. $x_{i}$ and $x_{j}$ are the independent variables, and ε is the error [23].

### 2.5. Artificial Neural Networks

Artificial neural networks (ANNs) can extract patterns of essential processes from past data and use this acquired knowledge to predict outcomes in new situations. These models efficiently grasp the complex mathematical relationships between input and output variables, enabling them to make effective predictions and adapt to varied conditions. ANN models have the power to predict any complex system on the basis of its architecture [34,35]. Neural network architectures typically involve one or more hidden layers, along with input and output layers, with the specific number being influenced by the experimental nature.

One of the fundamental characteristics of neural networks is their capacity to transform input data into corresponding outputs through a series of internal computational processes [36,37].

**Figure 2:**
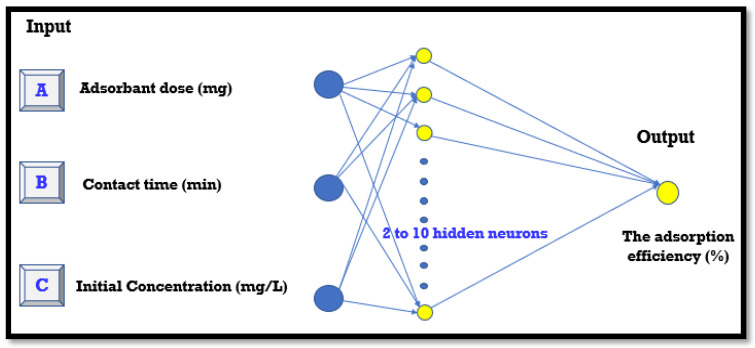
ANN architecture for the adsorption of phosphate ions onto the engineered bio-composite.

In this work, an artificial neural network (ANN) was employed to model the adsorption of phosphate ions using bio-composite beads. Key physicochemical factors, including adsorbent dose, contact time, and initial phosphate concentration, were investigated. The experimental data for the input variable were derived from the central composite design. Utilizing MATLAB 7 software, the ANN model was subjected to a training phase, followed by a validation process to assess its performance. The network structure included an input layer, a hidden layer of neurons, and an output layer. The input parameters were adsorbent dose, contact time, and initial phosphate ion concentration, while the output layer consisted of adsorption efficiency (%), as shown in Figure 2.

## 3. Results and Discussion

### 3.1. Characterization of Bio-Composite Beads

**Figure 3:**
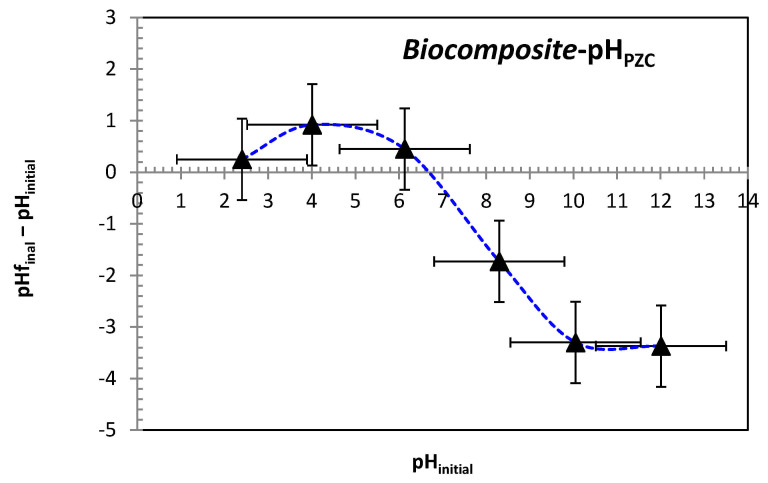
The point of zero charge (pH_PZC_) of bio-composite beads.

In order to establish the point of zero charge (pH_PZC_), six separate flasks were filled with 50 mL of 0.01 M NaCl solution, with pH values ranging between 2 and 12. An optimal mass of adsorbent was introduced into each flask, and the pH was adjusted using HCl and NaOH solutions (0.1 M). The flasks were stirred at room temperature for 48 h, after which the final pH of the solutions was registered. [38]. The pH at which the initial and final pH values intersect (Figure 3) was identified as the point of zero charge (pHₚzc), determined to be 6.7 for the bio-composite beads. This indicates that the adsorbent surface carries a positive charge at pH values below 6.7, and becomes negatively charged at pH values above this threshold.

Scanning electron microscopy (SEM) allows for the examination of grain morphology and the estimation of their approximate size, providing insight into the arrangement of grains, fibers, and fiber-like structures within the processed material.

**Figure 4:**
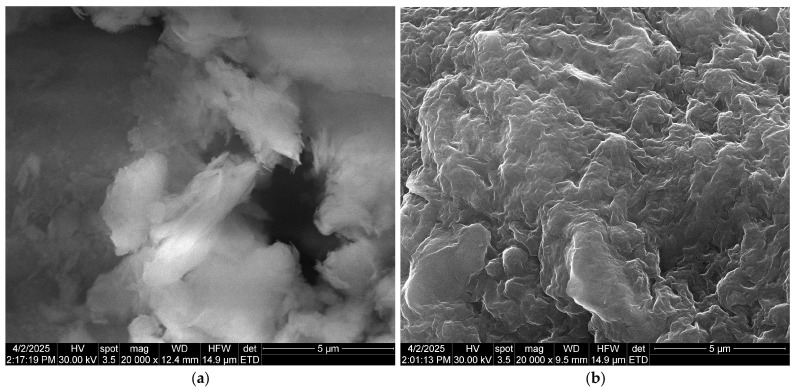
SEM images of (**a**) natural clay and (**b**) alginate–carrageenan–clay composite beads.

The bio-nanocomposite was characterized using SEM coupled with energy-dispersive X-ray spectroscopy (EDX) (Inspect F50, National University of Science and Technology Politehnica of Bucharest, Bucharest, Romania). SEM images depicting the natural clay microparticles (bentonite) and the studied bio-nanocomposite are shown in Figure 4. The incorporation of alginate and iota-carrageenan into the natural clay material induces significant changes in its textural morphology, as one can see in the scanning electron microscopy (SEM) images (Figure 4). Prior to modification, the natural clay surface appeared smooth and uniform (Figure 4a). However, after integration, it exhibited increased roughness and irregularity (Figure 4b). The increased surface roughness seen in SEM analysis significantly contributes to improving the adsorption capacity of materials intended for phosphate ion removal.

**Figure 5:**
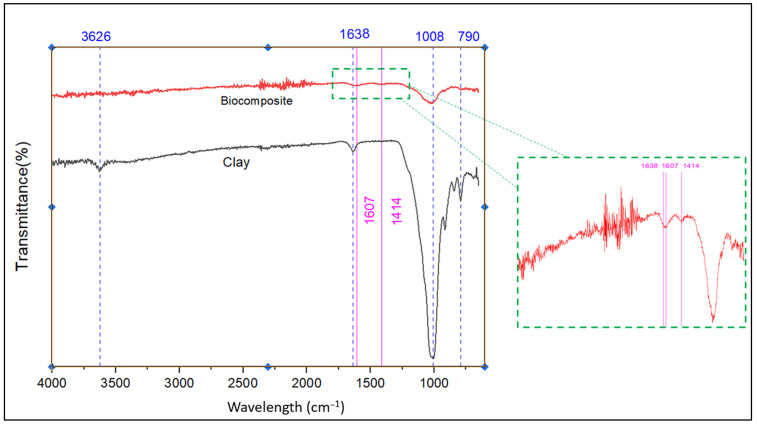
Fourier-transform infrared spectroscopy (FTIR) spectrum of bentonite natural clay and the engineered bio-composite.

Figure 5 displays the IR spectra of both natural clay and the engineered bio-composite. FT-IR spectroscopy was utilized to analyze their structural characteristics and surface functional groups.

As illustrated in Figure 5, the comparative FTIR spectrum between the natural clay and the engineered bio-composite, supported by EDX elemental data, highlights the structural changes resulting from the formation of the bio-composite. The disappearance of the band at 3626 cm^−1^, associated with the O–H vibrations of the octahedral alumina sheets, indicates the disruption of the clay structure, confirmed by the absence of aluminum in the bio-composite (6.12% Al in the clay). The strong intensity of the bands at 1607 and 1414 cm^−1^ in the bio-composite reflects the introduction of aromatic or carboxylate groups from the organic matter, corroborated by the notable increase in carbon content (49.2% vs. 8.64% in the clay). Meanwhile, the significant decrease in silicate bands at 1008 and 790 cm^−1^, as well as the reduction in silicon content (7.76% vs. 26.04%), suggests encapsulation of the mineral phase by the organic matrix. The exclusive presence of calcium (3.77%) in the bio-composite could indicate the addition of fillers or ions promoting crosslinking, while the consistent chlorine content (≈2%) does not contribute any notable spectral changes. These results confirm the formation of a hybrid bio-composite, where the incorporation of organic components significantly alters the initial mineral structure, modifying both the chemical composition and the vibrational signatures of the material.

The FT-IR spectrum of pure alginate (Figure S1) provided a clearer description of the main functional groups that contribute to the formation of the bio-composite. The spectrum shows key functional groups involved in interactions with natural clay and carrageenan. The broad band at ~3244 cm^−1^ corresponds to –OH groups, enabling hydrogen bonding. Peaks at ~1594 and ~1409 cm^−1^ are assigned to –COO^−^ groups, which can form ionic interactions with metal cations in clay and sulfate groups in carrageenan. The ~1020 cm^−1^ band (C–O–C) may participate in dipole interactions. The presence of hydroxyl and carboxylate groups makes alginate a suitable matrix for physical entanglement, hydrogen bonding, and ionic interactions with both clay (via surface hydroxyl groups and metal ions) and carrageenan (via sulfate groups). These interactions enhance the structural integrity and functional performance of the hybrid beads in phosphate adsorption.

**Figure 6:**
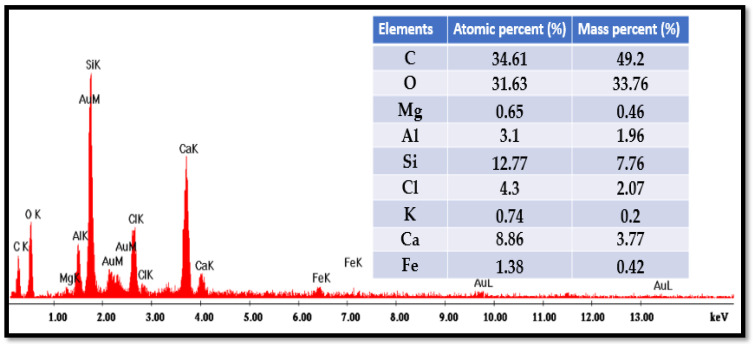
EDX analysis of alginate–carrageenan–clay composite beads.

The elemental composition of the bio-composite beads was examined using scanning electron microscopy (SEM) combined with energy-dispersive X-ray spectroscopy (EDX). The analysis was performed with a Quanta Inspect F50 system (FEI Company, Eindhoven, Netherlands). The EDX analysis of bio-nanocomposite beads is shown in Figure 6.

The energy-dispersive X-ray (EDX) analysis of the bio-nanocomposite beads reveals their atomic composition, with oxygen (O), carbon (C), silicon (Si), and calcium (Ca) accounting for approximately 31.63%, 34.61%, 12.77%, and 8.86%, respectively. The presence of calcium (around 1% of the atomic fraction) is attributed to the addition of alginate, which naturally incorporates calcium ions from the marine environment. Additionally, the elevated oxygen content and the presence of calcium in the EDX spectra validate the successful incorporation of sodium alginate and iota-carrageenan into the natural clay matrix.

### 3.2. Factors Controlling the Adsorption of Phosphate Ions

**Figure 7:**
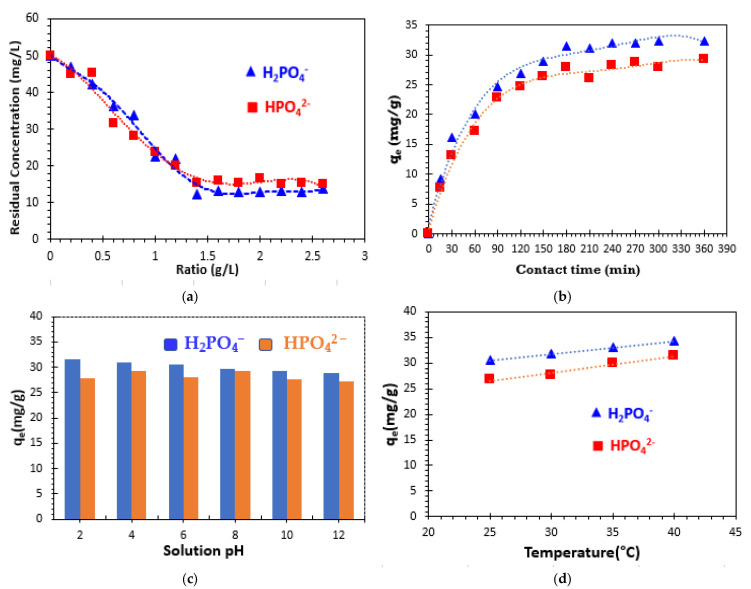
Effect of factors controlling the adsorption of phosphate ions: (**a**) ratio; (**b**) contact time; (**c**) initial pH; and (**d**) temperature.

The effect of adsorbent dose on the adsorption of phosphate ions by bio-composite beads was examined. This study involves determining the mass/volume ratio (R = m/V) that would lead to the maximum removal of phosphate ions using a minimum quantity of the adsorbent. A volume of 50 mL for each 50 mg/L of phosphate ion solution was placed in contact with various masses of bio-composite beads. Figure 7a illustrates the variation in the amount of phosphate ions adsorbed as a function of the adsorbent ratio. The results obtained demonstrate that adsorption capacity increases with increasing bio-composite mass, stabilizing at an optimum ratio of 1.4 g/L, which corresponds to the minimum ratio required for maximum adsorption after a contact time of 12 h. Above this optimum value, an equilibrium plateau is reached. Moreover, the presence of a larger mass of the adsorbent in solution leads to an increase in the availability of exchangeable sites, resulting in a greater number of active sites for phosphate ion uptake [39,40,41]. The equilibrium plateau may be attributed to the overlapping of adsorption sites due to the crowding of absorbing particles. This phenomenon can be attributed to the fact that a large amount of the adsorbent leads to particle agglomeration, resulting in a reduction in the total adsorption surface area and consequently decreasing the amount of adsorbate per unit mass of adsorbent [12,42]. The results of this study suggest that the increase in phosphate removal percentage is linked to an increase in surface area and adsorption sites as a consequence of the increase in adsorbent mass. At a ratio of 1.4 g/L, the percentage of phosphate removal is around 75.56% for H_2_PO_4_^−^ and 68.99% for HPO_4_^2−^ and remains almost constant.

Thus, increasing the amount of adsorbent makes a large number of sites available, resulting in increased adsorption [43].

The influence of contact time on the adsorption of the phosphate ions studied (H_2_PO_4_^−^ and HPO_4_^2−^) was investigated using the initial concentration of 50 mg/L. The effect of contact time on the removal of the phosphate ions from a solution at room temperature (23 ± 2 °C) and initial pH (equal to 7.27 for the HPO_4_^2−^ solution and 5.09 for the H_2_PO_4_^−^ solution) is illustrated in Figure 7b. It is observed that the adsorbed uptake of phosphate ions on the surface of bio-composite beads increases with prolonged contact time. The adsorption process is particularly rapid within the initial 150 min. This result can be explained by the high initial availability of free active sites, which facilitates the adsorption process [43,44,45,46]. Subsequently, adsorption equilibrium is attained after approximately 180 min of contact time. This result indicates a stable state, possibly attributed to the almost total occupation of the adsorption sites available [46]. Given the duration of the study, 180 min was selected as the optimal contact time for subsequent adsorption experiments. Figure 7b indicates that, at equilibrium, the amount of phosphate adsorbed by the composite beads can reach 31.48 mg/g for H_2_PO_4_^−^ and 27.82 mg/g for HPO_4_^2−^ in aqueous solutions.

A similar study was conducted by Nouaa et al. (2023) on phosphate ion removal using a bio-composite based on anionic HDL clay and alginate. This study is characterized by two steps, the first of which is rapid during the first 60 min, attributed to the abundant availability of free sites and therefore a higher probability of adsorption [5]. The second slow stage is attributed to a decrease in the availability of free sites, as most of the active sites are already occupied by adsorbed molecules. According to Parvin et al. (2019), the adsorption process is rapid in the first step due to the availability of vacant active sites, while in the second step, active sites are saturated and the adsorption capacity becomes constant [47].

The effect of solution pH was then manipulated across the pH range of 2 to 12 by the addition of 0.1 M HCl or 0.1 M NaOH solutions [12,15,46]. To evaluate the impact of solution pH, an experimental procedure was conducted. A quantity of 0.07 g of bio-composite beads was introduced into multiple glass vials, each containing 50 mL of phosphate solution. The pH of these solutions was adjusted within a range of 2 to 12. Figure 7c illustrates the phosphate adsorption behavior of clay/alginate/carrageenan composite beads as a function of the initial solution pH. The adsorption capacity remained constant at all pH values (between 28.93 and 31.48 mg/g for H_2_PO_4_^−^ ions and between 27.24 and 29.36 mg/g for HPO_4_^2−^ ions). The results suggest that the phosphate removal efficiency of the bio-composite beads in this study remains unaffected by pH variations in the solution between 2 and 12 under the specified experimental conditions.

Our findings align with those documented by Nouaa et al. (2023) and Han, Yong-Un, et al. (2011) [5,48]. The engineered bio-composite exhibits resistance to variations in solution pH, primarily due to the stabilizing effects of the alginate gel and iota-carrageenan structures. These components create a protective environment around the natural clay particles, mitigating the impact of pH variations on adsorption capacity. This stability enhances the efficiency of phosphate ion removal across different pH conditions [5].

The temperature of the phosphate ion solution can significantly influence the entire adsorption process, particularly the adsorption capacity. The impact of temperature on the adsorption of phosphate ions by the engineered bio-composite adsorbent was investigated within the temperature range of 25 °C to 40 °C, as depicted in Figure 7d. It was observed that the adsorption capacity improves with increasing temperature, suggesting that the adsorption process is endothermic in nature.

### 3.3. Adsorption Kinetics

Various kinetic adsorption models have been utilized to describe the mechanism of the adsorption of inorganic pollutants by bio-composite adsorbents [5,11,12,15]. To determine the most suitable model that best represents the experimentally obtained kinetic data, different approaches, including PFO, PSO, IPD, and the Elovich model, were applied. The selection of the optimal model was based on the assessment of the correlation coefficients (R^2^).

**Figure 8:**
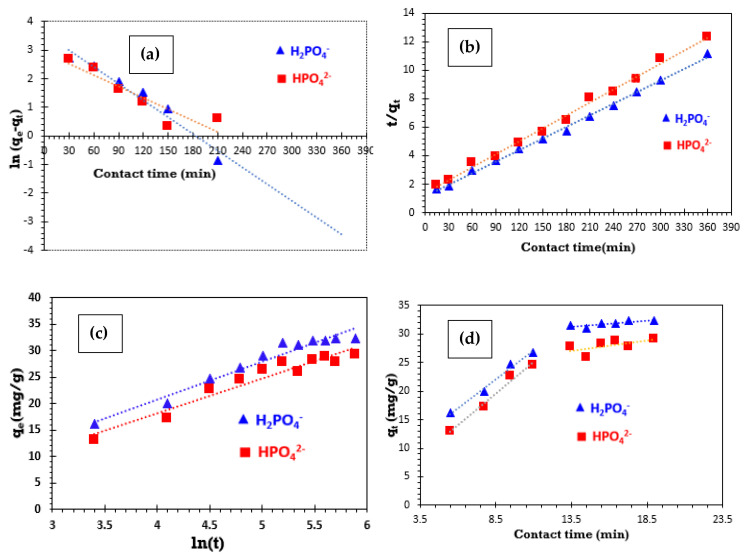
Kinetic models for the adsorption of phosphate ions on bio-composite beads: (**a**) PFO model; (**b**) PSO model; (**c**) Elovich model; and (**d**) IPD model.

In the case of PFO, plotting ln(*q_e_* − *q_t_*) against contact time (t) yields a linear relationship, characterized by a slope of -k_1_ and an intercept of ln(*q_e_*) (Figure 8a).

Table 4 presents the values for the theoretical adsorption capacity (*q_e_, _Theo_*), the rate constant associated with the PFO kinetics model (*k*_1_), and the correlation coefficient (R^2^). As shown in Figure 8a and Table 4, when applying the pseudo-first-order kinetic model, the correlation coefficient values are relatively low, and the equilibrium adsorption capacity (*q_e_, _Theo_*, mg/g) calculated from this equation is lower than the experimentally determined value (*q_e_*, _*Exp*_, mg/g). It could be seen that the correlation coefficients R^2^ of the pseudo-first-order model were 0.956 for H_2_PO_4_**^−^** ions and 0.839 for HPO_4_^2−^ ions. Under these conditions, the pseudo-first-order model does not adequately describe the adsorption kinetics of phosphate ions from aqueous solutions onto bio-composite bead adsorbents.

Figure 8b illustrates the PSO adsorption kinetics model for phosphate ions, with the corresponding kinetic parameters detailed in Table 4. The PSO kinetic model exhibits a correlation coefficient close to 1, indicating strong agreement with experimental data. Additionally, the theoretically adsorbed quantity (*q_e_*, _*Theo*_) closely aligns with the experimentally observed values, measuring 36.90 mg/g for H_2_PO_4_^−^ ions and 33.003 mg/g for HPO_4_^2−^ ions.

The correlation coefficient values (R^2^) derived from the q_t_ versus ln (t) plot using the Elovich model for phosphate ion adsorption on bio-composite beads (Figure 8c) are consistently lower than those obtained with the PSO kinetic model, as shown in Table 4. This difference in R^2^ values suggests that the Elovich model is less effective in providing an accurate representation of the adsorption process of phosphate ions onto bio-composite beads.

The graphical representation of dependence q_t_ versus t^1/2^, obtained for the adsorption of phosphate ions on bio-composite beads used in this study (Figure 8d) does not go through the origin, and two separated regions were obtained.

The deviation of the straight-line plots from the origin suggests that intra-particle diffusion is not the primary rate-controlling step; rather, boundary layer diffusion plays a significant role in the adsorption process. Additionally, the higher rate constant values obtained from this model, compared to the pseudo-second-order model, further support the notion that intra-particle diffusion does not limit the rate of phosphate ion adsorption onto bio-composite beads. The observed multi-linearity indicates that the adsorption process involves multiple elementary steps. The initial phase corresponds to the rapid transfer of phosphate ions from the bulk solution to the adsorbent surface, while the second phase involves intra-particle diffusion within the adsorbent.

Finally, the results confirm that the PSO kinetic model has a good correlation with the experimental data for the adsorption process. A similar study by Wenyun et al. (2018) showed that the adsorption of several organic pollutants tested using gelatin/bentonite composite beads followed the PSO kinetic model [49].

Another similar study by Barrak et al. (2014) on the removal of copper ions from an aqueous medium using sodium alginate encapsulated Moroccan clay as an eco-friendly method showed that the adsorption kinetic data were well-fitted to the PSO model, and the correlation coefficient was notably high [11].

### 3.4. Adsorption Isotherms

**Figure 9:**
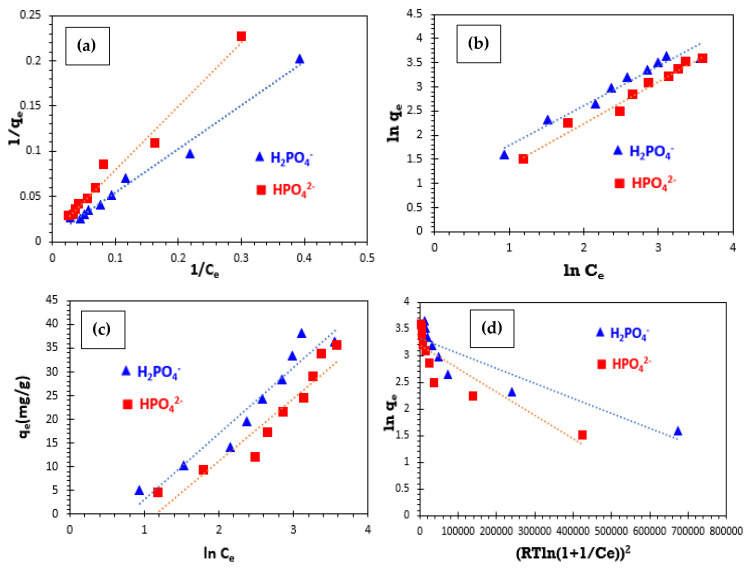
Isotherm models at 25 °C: (**a**) Langmuir model, (**b**) Freundlich Model, (**c**) Temkin model, and (**d**) Dubinin–Radushkevich (D–R) Model.

The investigation into adsorption isotherms provided a detailed understanding of the interactions between pollutants and adsorbent materials, highlighting the precision with which adsorption processes align with mathematical models and the significance of the parameters involved. Isotherm experiments were conducted under optimal conditions, with initial concentrations ranging from 10 to 90 mg/L. Several adsorption models were evaluated, including Langmuir, Freundlich, Temkin, and Dubinin–Radushkevich (D-R). Graphical representations of the Langmuir (1/q_e_ vs. 1/C_e_), Freundlich (ln q_e_ vs. ln C_e_), Temkin (q_e_ vs. ln C_e_), and Dubinin–Radushkevich (D-R) (ln qe vs. ɛ^2^) models are presented in Figure 9. These graphs illustrate the adsorption behavior of phosphate ions on bio-composite bead adsorbents, providing valuable insights into the interaction dynamics between inorganic pollutants and adsorption materials. The Langmuir adsorption isotherm is employed to describe monolayer adsorption taking place solely on the uniform surface of the adsorbent and was linearly transformed, as shown in Figure 9a. The values for the adsorption capacity (q_L_), Langmuir constant (K**_L_**), and correlation coefficient (R^2^) are shown in Table 5.

The maximum adsorption capacity value (q_L_, mg·g^−1^) obtained at 25 °C was 140.84 mg·g^−1^ for H_2_PO_4_^−^ ions and 105.26 mg/g for HPO_4_^2−^ ions.

The Langmuir isotherm is characterized by a dimensionless parameter called the separation factor (R_L_), which serves as an indicator for determining the favorability of an adsorption system. When R_L_ falls within the range 0 < R_L_ < 1, the adsorption process is considered favorable. If R_L_ exceeds 1, the process is unfavorable, while R_L_ = 1 indicates linear adsorption behavior. An adsorption process is deemed irreversible when R_L_ equals 0. The value of RL is determined using the corresponding mathematical equation [8,12,15].

(4) $$R_{L}=\frac{1}{1+K_{L}C_{o}}$$

In this context, K_L_ (L·mol^−1^) represents Langmuir’s constant, while C_0_ (mol·L^−1^) denotes the highest initial concentration.

In this study, the determined values of the separation factor (R_L_) ranged between 0 and 1, specifically 0.986 for H_2_PO_4_^−^ ions and 0.987 for HPO_4_^2−^ions. These results confirm that the adsorption of phosphate ions onto bio-composite bead particles was favorable.

The Freundlich isotherm model suggests that adsorption occurs in multiple layers and follows a heterogeneous and reversible process, without reaching saturation at high concentrations [15,50]. The slope and intercept of the ln q_e_ vs. ln C_e_ plot were utilized to determine the isotherm constants n and K_F_ (Figure 9b). Table 5 presents the values of the Freundlich constants (K_F_ and n) along with the correlation coefficients (R^2^) for phosphate ions. The adsorption process is considered favorable when the value of 1/n falls between 0 and 1, while a value of 0 indicates a higher degree of surface heterogeneity [51,52]. Furthermore, if n is below 1, the process is classified as chemical adsorption, whereas when n is greater than 1, it corresponds to physical adsorption [53].

The Temkin adsorption isotherm model is founded on the heat of ionic adsorption resulting from interactions between the adsorbate and the adsorbent. The corresponding Temkin isotherm plot is depicted in Figure 9c. Meanwhile, the Dubinin–Radushkevich (D-R) isotherm is commonly applied to describe the adsorption mechanism, incorporating a Gaussian energy distribution across a heterogeneous surface (Figure 9d). This model is particularly useful for distinguishing between physical and chemical adsorption processes based on the average adsorption energy (E), which is determined using the relevant equation:

(5) $$E=\frac{1}{{\left(2K_{D}\right)}^{1/2}}$$

When the value of E falls between 8 and 16 kJ·mol^−1^, the adsorption mechanism is considered chemical. In contrast, if E is lower than 8 kJ·mol^−1^, the process is classified as physisorption. The obtained results and computed parameters are presented in Table 5.

These results indicate that the correlation coefficients (R^2^) are significant for both the Langmuir and Freundlich isotherm models. The experimental data align precisely along a straight line, with correlation coefficients approaching 1. The computed separation factor (RL) falls within the range of 0 to 1, confirming the favorable adsorption of phosphate ions onto bio-composite beads. The Freundlich isotherm model presents a 1/n value below 1, meaning n exceeds 1, which suggests that the adsorption process is predominantly physical. In contrast, the Temkin and Dubinin–Radushkevich (D-R) isotherm models exhibit lower correlation coefficients compared to those observed for the Langmuir and Freundlich models. This comparison suggests that the adsorption of phosphate ions is promoted by the Langmuir and Freundlich models, indicating diversity in adsorption sites. Nouaa et al. (2023) performed a similar study on the adsorption of phosphate ions using LDH/alginate composite beads and showed that the Langmuir and Freundlich isotherm models were in good agreement with the experimental data as high R^2^ values exceeding 0.97 were obtained [5].

### 3.5. Thermodynamic Study

The thermodynamic analysis was conducted to gain deeper insights into the characteristics and mechanisms of the phosphate ion removal process using alginate–carrageenan bio-composite beads as a bio-adsorbent. The thermodynamic parameters, including ΔG° (kJ/mol), ΔH° (kJ/mol), and ΔS° (J/K·mol), were determined based on specific equations referenced in previous studies [12,15,39,54].

(6) $$∆G{}^{\circ}\;=\;-RTLn\left(K_{d}\right)$$

(7) $$ln\left(Kd\right)=-\frac{∆H{}^{\circ}}{RT}+\;\;\frac{∆S{}^{\circ}}{R}$$

(8) ∆G°  = ∆H°−T∆S°

where T is taken as the absolute temperature in Kelvin, R represents the universal gas constant (8.314 J/mol^−1^K^−1^), and K_d_ (L·mol^−1^) is the distribution coefficient. The determination of the thermodynamic parameters was carried out using the information provided in Figure S2.

Table 6 shows the results for thermodynamic parameters. It is acknowledged that the heat of adsorption (ΔH°) values necessary for physisorption generally fall within the range of 20 kJ·mol^−1^ or less. In contrast, the values required for chemisorption tend to be higher, typically ranging from 80 to 200 kJ·mol^−1^ [55].

The positive ∆H° values indicate that the adsorption is endothermic and mainly driven by physical forces, which is consistent with experimental findings. Negative ∆G° values confirm the spontaneity of the process, while the positive ∆S° values reflect increased randomness at the solid–liquid interface due to the attachment of ion-organic species onto the bio-nanocomposite surface [56,57].

### 3.6. Design of Experiments for BBD-RSM

#### 3.6.1. Analysis of Variance and Residuals

Adsorption efficiency (%) was optimized using the Box–Behnken design combined with response surface methodology (BBD-RSM). A quadratic polynomial model was applied, showing good agreement between adjusted and predicted R^2^ values. The model’s statistical significance and reliability were further supported by ANOVA results.

ANOVA was employed to evaluate the statistical significance of the interactions between the independent variables and the response in the adsorption model [58]. As shown in Table 7, the model incorporating adsorbent dose (A), contact time (B), and initial H_2_PO_4_^−^ concentration (C) was found to be statistically significant.

The data in Table 7 indicate that the adsorbent dose (A), contact time (B), their interaction (AB), the quadratic term of the adsorbent dose (A^2^), and the quadratic term of the adsorbent dose (B^2^) are the key parameters significantly influencing the adsorption efficiency (%).

The initial concentration factor (A), adsorbent dose versus initial concentration (AC), and contact time versus initial concentration (BC) do not have a significant impact, as the *p*-values exceed 0.005. In the ANOVA analysis, a model is considered significant when the *p*-value is below 0.05, emphasizing the importance of the model [37,59]. Additionally, the model’s F-value of 26.45 further confirms its significance [35]. Adeq precision estimates the signal-to-noise ratio, which is greater than 4. Since the ratio is 15.4198, the signal is sufficient. This model may be used to explore the design space (Table 7).

The accuracy of the developed models is demonstrated by the correlation coefficients (R^2^). In this study, the R^2^ value of 0.9714 confirms the excellent alignment of the data. This value indicates that the proposed models account for 97.14% of the variance in adsorption efficiency (%). The large correlation coefficient R^2^ (0.9714) and the adjusted correlation coefficient $R_{aj}^{2}$ (0.9347) confirm the adequacy of the conditions for checking the proposed model (refer to Table 8).

The equation for the quadratic model obtained for the adsorption process concerning the input factors is presented in the following equation:

Y (Adsorption efficiency (%)) = 74.85 + 19.34 A + 21.68 B − 0.9731 C + 10.19 AB − 2.50 AC + 0.0347 BC − 20.80 A^2^ -21.81 B^2^ − 4.80 C^2^

**Figure 10:**
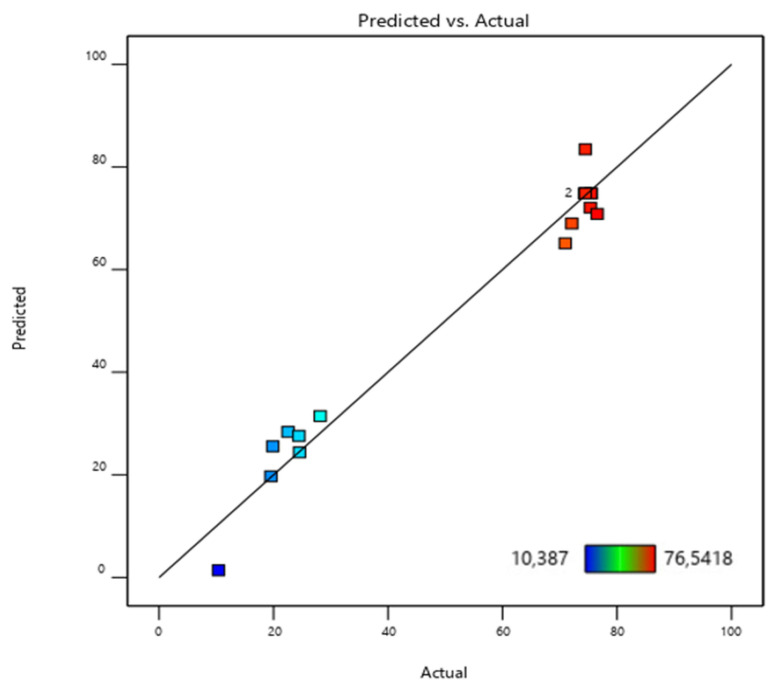
Comparison of the experimental and BBD-RSM-predicted adsorption efficiency (%) response.

The model equation may be used to make predictions regarding the responses for a given factor level. Figure 10 shows the normal distribution with standard residuals. The residuals are normally distributed along the line.

It is apparent that the straight line drawn among these points encompasses almost all of the data, and an approximate normality is observed.

#### 3.6.2. Graphical Representation of Response Surfaces

**Figure 11:**
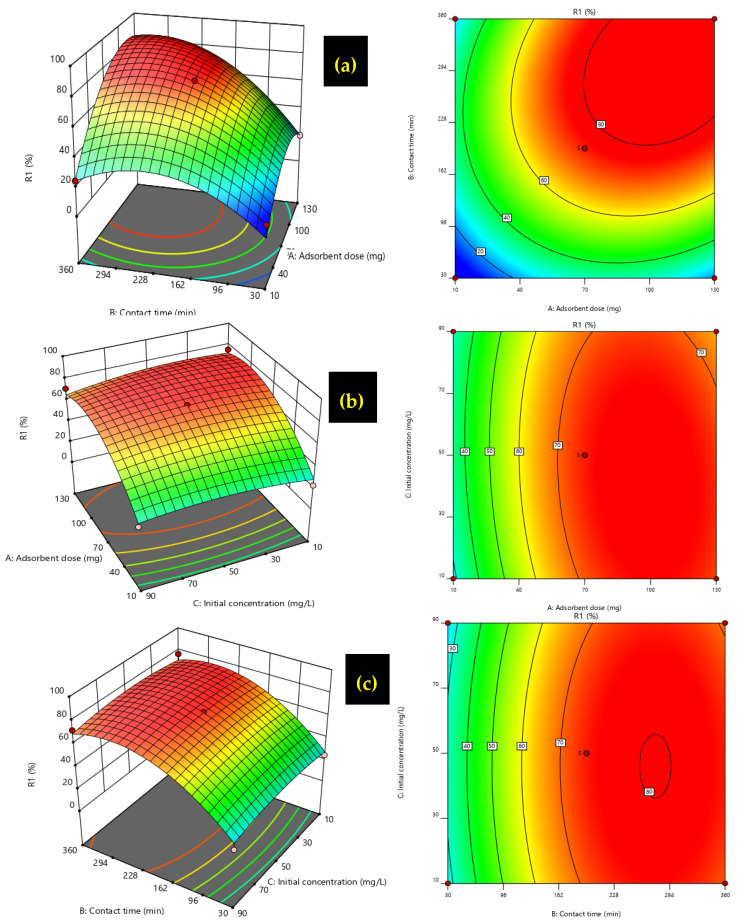
The 3D response object and contour plots illustrating the interaction between: (**a**) adsorbent dosage versus contact time, (**b**) adsorbent dosage versus initial concentration, and (**c**) contact time versus initial concentration.

Contour plots (Figure 11) were generated by Design Expert 12 software to investigate the effect of the interactions of the three variables tested on adsorption efficiency Y (%). These plots provide a visual interpretation of the dependence of the response behavior on the changing variables. The 3D surface and contour plots illustrate the combined effect of two parameters on adsorption performance while keeping the third parameter constant. Generated from the regression model, the 3D response surfaces highlight the interaction between variables and their influence on the efficiency of the adsorption process. These graphical plots help determine the optimal conditions by statistically analyzing different combinations of variables. The most significant correlations for a high adsorption rate are adsorbent dose versus contact time (Figure 11a), adsorbent dose versus initial concentration (Figure 11b), and contact time versus initial concentration (Figure 11c). These graphs indicate that to obtain a high H_2_PO_4_^−^ ion adsorption efficiency (R_1_ (%)), the adsorbent dose (A) and the contact time (B) should be maintained at a high level. The initial H_2_PO_4_^−^ ion concentration remains insignificant.

#### 3.6.3. Optimization Using the Desirability Function

The desirability function was employed to identify optimal conditions that ensure both high adsorption efficiency and acceptability. Table 8 and Figure S3 provide a summary of the criteria, constraints, and optimal solutions achieved for the process. Consequently, the ideal conditions predicted for the process include an adsorbent dose equal to 88.3316 mg, a contact time equal to 76.9405 min, and an initial concentration of H_2_PO_4_^−^ ions equal to 56.977 mg/L.

### 3.7. Artificial Neural Network Modeling (ANN)

**Figure 12:**
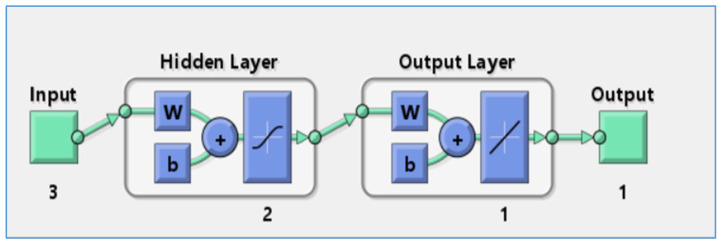
Optimum ANN structure (3-2-1).

To establish the most effective ANN model architecture, the highest R^2^ value obtained during testing was taken into account. The training process was refined using the standard backpropagation algorithm. The optimal topology for H_2_PO_4_^−^ ion adsorption by alginate–carrageenan–clay composite beads was determined to be 3-2-1, demonstrating a strong R^2^ value [60]. As illustrated in Figure 12, the optimized ANN model incorporates two neurons in the hidden layer and achieves the highest correlation coefficient across all datasets, reinforcing the robust relationship between experimental data and predicted outcomes.

**Figure 13:**
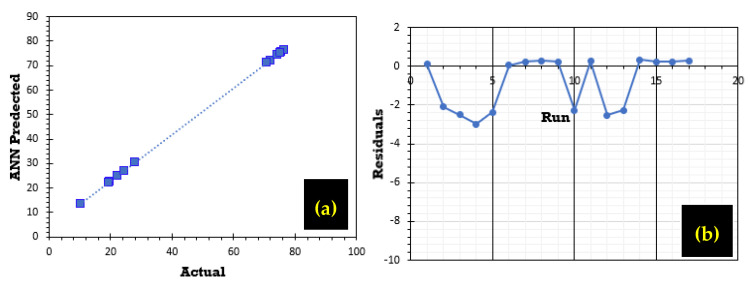
(**a**) Comparison of experimental and ANN-predicted adsorption capacities and (**b**) residual study of the ANN prediction.

The graphs in Figure 13a,b show a strong correlation, illustrating the close alignment between experimental values and those predicted by the ANN model. Figure 13a highlights the agreement between predicted and experimental phosphate ion adsorption efficiency. Furthermore, the residual analysis illustrated in Figure 13b for the predicted adsorption rate R (%) indicates a random pattern distributed around zero.

**Figure 14:**
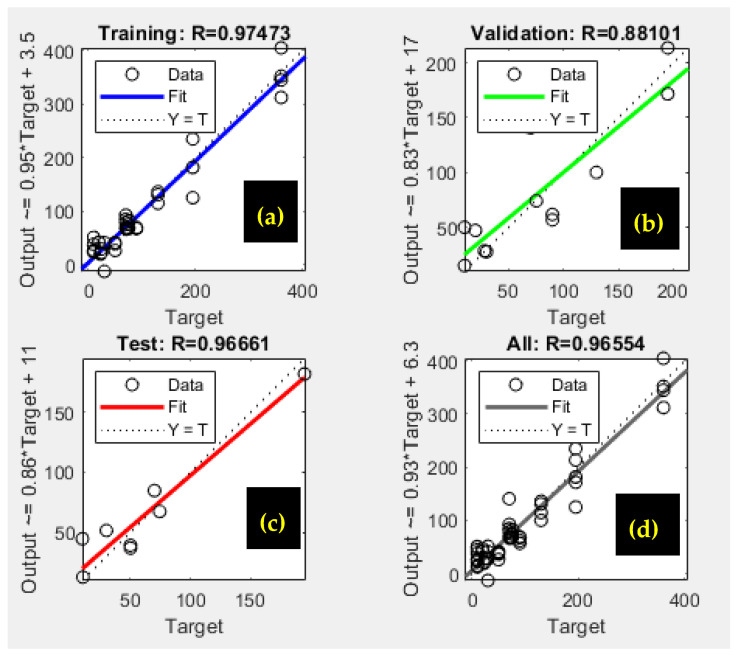
ANN correlation graphs depicting (**a**) training, (**b**) validation, (**c**) testing, and (**d**) overall network performance.

Figure 14 illustrates the correlation between the experimental results and the values predicted by the neural network model. The solid line denotes the ideal fit, indicating where the data points would lie if the model’s predictions perfectly matched the experimental data. In contrast, the dotted line represents the best linear fit to the scattered data, highlighting the model’s effectiveness in approximating the observed outcomes [35,61].

The correlation coefficient (R^2^ = 0.974) highlights the model’s effectiveness in predicting phosphate ion adsorption on bio-composite beads. As shown in Figure 14a, the ANN model demonstrated a strong correlation between the trained data and the optimized structure, performing relatively well during validation and testing despite some data dispersion [35,37,61] (Figure 14b,c).

Overall, the model provided satisfactory results for the batch adsorption experimental dataset, as presented in Figure 14d.

### 3.8. Adsorption Mechanisms

**Figure 15:**
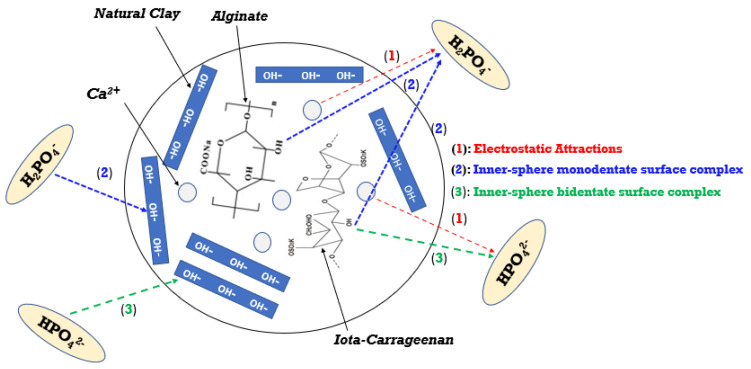
Mechanisms of adsorption for H_2_PO_4_^−^ and HPO_4_^2−^ ions on alginate–carrageenan–clay bio-composite beads.

The adsorption mechanism of the developed bio-composite for H_2_PO_4_^−^ and HPO_4_^2−^ ions is presented in Figure 15. Phosphate ion removal primarily involves three mechanisms: electrostatic attraction, monodentate inner-sphere surface complexation, and bidentate inner-sphere surface complexation. These interactions play a crucial role in understanding the adsorption behavior of phosphate onto bio-composite materials. [12,62]. Electrostatic attraction is generally an essential initial step, given that the negatively charged anionic species H_2_PO_4_^−^ and HPO_4_^2−^ interact with positively charged (protonated) sites on the surface of the bio-composite. This interaction is expected, as cationic elements such as Ca^2+^ and Al^3+^ play a specific role in facilitating this mechanism [12]. The formation of inner-sphere surface complexes (M–O–P) indicates a direct chemical interaction between phosphate ions and the surface of the bio-composite.

### 3.9. Comparison with Published Data

To put our adsorbent into context in comparison to materials utilized for the elimination of inorganic pollutants from aqueous solutions, the maximal adsorption capacity of the alginate–carrageenan–clay composite was then contrasted with reports from the literature containing the maximal values of adsorption capacities (q_max_, mg/g) for other adsorbents. A comparative overview of phosphate ion adsorption by various referenced adsorbents is provided in Table 9. The q_max_ value obtained from this work was found to be comparable to published values for other materials, therefore showing that the bio-nanocomposite studied here is of high potentiality for applications in wastewater treatment processes.

## 4. Conclusions

This study focuses on the synthesis of an innovative hydrogel bio-composite using natural clay, sodium alginate (Na-AL), and iota-carrageenan as adsorbents for phosphate ions from aqueous solutions. Various characterization techniques, including Fourier-transform infrared (FTIR) spectroscopy, scanning electron microscopy coupled with energy dispersive X-rays (SEM-EDX), and point zero charge (PZC) determination, were used to analyze the adsorbents.

The adsorption process was examined in relation to key influencing factors such as adsorbent dosage, contact time, solution pH, and temperature. Four isotherm models and four kinetic models were applied to assess phosphate ion removal on the prepared bio-composite. The findings indicate that the second-order kinetic model provides the most accurate description of the adsorption process. The correlation coefficients (R^2^) in both the Langmuir and Freundlich models were found to be very close to 1, demonstrating that these models effectively characterize phosphate adsorption by accounting for a variety of adsorption sites. The bio-composite achieved a maximum adsorption capacity of 140.84 mg/g for H_2_PO_4_^−^ ions and 105.26 mg/g for HPO_4_^2−^ ions in batch experiments.

Thermodynamic analysis indicates that the adsorption process is both endothermic and governed by physical interactions, as evidenced by the positive ∆H° values, which are consistent with the experimental findings. The spontaneity of the process is confirmed by the negative ∆G° values, while the positive ∆S° values imply increased randomness at the solid–liquid interface during the adsorption of phosphate ions onto the engineered bio-composite. Optimization of key parameters, including the adsorbent dosage, contact time, and initial concentration, was achieved using the Box–Behnken design within the response surface methodology (BBD-RSM). The reliability of the developed models is demonstrated by an R^2^ value of 0.9714, indicating a highly accurate fit to the data and explaining 97.14% of the variance in adsorption efficiency.

Finally, the adsorption behavior of phosphate ions onto bio-composite beads was analyzed using an artificial neural network (ANN) to predict process efficiency. The ANN model demonstrated strong predictive capabilities, achieving a significant correlation coefficient of 0.974 between model predictions and experimental results.

## Figures and Tables

**Table 1:** The various forms of kinetic models.

Kinetic Models	Non-Linear Form	Linear Form	Plots
***PFO***	$q_{t}=q_{e}\left(1-e^{-k_{1}t}\right)$	$ln\left(q_{e}-q_{t}\right)=lnq_{e}-k_{1}t$	*ln* (*q_e_ − q_t_*) = *f*(*t*)
***PSO***	$q_{t}=\frac{q_{e}^{2}k_{2}t}{1+(q_{e}k_{2}t)}$	$\frac{t}{q_{t}}=\frac{1}{k_{2}{q_{e}}^{2}}+\frac{1}{q_{e}}t$	$\frac{t}{q_{t}}=f(t)$
***IPD***	-	$q_{t}=K_{IPD}t^{1/2}+C$	$q_{t}=f(t^{\frac{1}{2}})$
***Elovich***	$\frac{dq_{t}}{dt}=\alpha \;e^{-\beta q_{t}}$	qt=ln(αβ)β+1β ln(t)	$q_{t}=f(lnt)$

**Table 2:** Two-parameter isotherm models.

Isotherm	Non-Linear Form	Linear Form	Plot
***Langmuir***	$\frac{q_{e}}{q_{L}}=\frac{K_{L}C_{e}}{1+K_{L}C_{e}}$	$\frac{C_{e}}{q_{e}}=\frac{1}{q_{L}K_{L}}+\frac{1}{q_{L}}\times C_{e}$	$\frac{C_{e}}{q_{e}}vsC_{e}$
		$\frac{1}{q_{e}}=\left(\frac{1}{q_{L}K_{L}}\right)\frac{1}{C_{e}}+\frac{1}{q_{L}}$	$\frac{1}{q_{e}}vs\frac{1}{C_{e}}$
		$q_{e}=q_{L}-(\frac{1}{K_{L}})\frac{q_{e}}{C_{e}}$	$q_{e}vs\frac{q_{e}}{C_{e}}$
		$\frac{q_{e}}{C_{e}}=K_{L}q_{L}-K_{L}q_{e}$	$\frac{q_{e}}{C_{e}}vs\;q_{e}$
		$\frac{1}{C_{e}}=K_{L}q_{L}\frac{1}{q_{e}}-K_{L}$	$\frac{1}{C_{e}}vs\frac{1}{q_{e}}$
***Freundlich***	$q_{e}=KC_{e}^{\frac{1}{n}}$	$lnq_{e}=lnK_{F}+\frac{1}{n_{f}}lnC_{e}$	$lnq_{e}vs\;lnC_{e}$
***Temkin***	$q_{e}=\frac{RT}{b}ln\left(K_{T}C_{e}\right)\;\;\;\;\;$	$q_{e}=B_{T}ln\left(K_{T}\right)+B_{T}ln\left(C_{e}\right)$ $B_{T}=\frac{RT}{b}\;\;$	$q_{e}vs\;lnC_{e}$
***Dubinin–Radushkevich***	$q_{e}=q_{m}exp(-K\varepsilon^{2})$	$lnq_{e}=lnq_{m}-K_{D}{ɛ}^{2}$ $\varepsilon =RTln(1+\frac{1}{C_{e}})$	$lnq_{e}vs\;{ɛ}^{2}$

**Table 3:** Range and levels of experimental parameters.

Variable	Name	Levels		
		−1	0	1
A	Adsorbent dose (mg/L)	10	70	130
B	Contact time (min)	30	195	360
C	Initial concentration (mg/L)	10	50	90

**Table 4:** Kinetic model parameters.

Kinetic Models	Parameters	Phosphate Ions	
		H_2_PO_4_^−^	HPO_4_^2−^
PFO			
	k_1_ (min^−1^)	0.019	0.013
	q_e cal_ (mg·g^−1^)	36.74	18.75
	R^2^	0.956	0.839
PSO			
	k_2_ (g·mg^−1^min^−1^)	0.00063	0.00067
	q_e cal_ (mg·g^−1^)	36.90	33.003
	R^2^	0.997	0.995
Elovich			
	α	2.417	1.873
	β	0.139	0.150
	R^2^	0.957	0.931
IPD			
Region I	k_PI_ (mg·g^−1^min^1/2^)	2.006	2.211
	c_I (mg/g)_	5.053	0.793
	R^2^	0.988	0.980
Region II			
	k_PII_ (mg·g^−1^min^1/2^)	0.210	0.352
	c_II_	28.46	22.32
	R^2^	0.720	0.397
Exp. adsorption capacity (q_e exp_, mg/g)		31.48	27.82

**Table 5:** Calculated parameters of the equilibrium isotherms using linear regression analysis.

Isotherms	Parameters	Phosphate Ions	
		H_2_PO_4_^−^	HPO_4_^2−^
Langmuir			
	K_L_ (L·mg^−1^)	0.014	0.013
	q_L_ (mg·g^−1^)	140.84	105.26
	R^2^	0.985	0.980
Freundlich			
	K_F_ (mg·g^−1^)	2.687	1.656
	1/n	0.812	0.866
	**R^2^**	0.956	0.978
Temkin			
	K_T_ (L·mg^−1^)	0.458	0.316
	b_T_ (J·mol^−1^)	178.07	187.19
	R^2^	0.923	0.962
Dubinin–Radushkevvich			
	K_D_ (mol^2^·J^−2^)	3 × 10^−6^	4 × 10^−6^
	q_m_ (mg·g^−1^)	28.03	24.33
	E (J·mol^−1^)	5 × 10^2^	5 × 10^2^
	R^2^	0.926	0.952

**Table 6:** Thermodynamic parameters for phosphate ion adsorption at 25 °C (C_0_ = 50 mg/L).

Ion	∆G° (kJ·mol^−1^)				∆H° (kJ·mol^−1^)	∆S° (J·K^−1^·mol^−1^)
	298 K	303 K	308 K	313 K		
H_2_PO_4_^−^	−12.98 ± 1.65	−13.48 ± 1.72	−14.16 ± 1.80	−14.56 ± 1.87	28.01 ± 1.20	134.06 ± 3.91
HPO_4_^2−^	−11.78 ± 4.90	−12.23 ± 5.00	−13.10 ± 5.10	−13.76 ± 5.20	28.84 ± 3.50	136.00 ± 11.47

**Table 7:** ANOVA analysis for the postulated model.

Source	Sum of Squares	df	Mean Square	F-Value	*p*-Value	
**Model**	11,454.50	9	1272.72	26.45	0.0001	significant
**A—Adsorbent dose**	2992.25	1	2992.25	62.19	<0.0001	significant
**B—Contact time**	3759.31	1	3759.31	78.13	<0.0001	significant
**C—Initial concentration**	7.57	1	7.57	0.1574	0.7034	
**AB**	415.17	1	415.17	8.63	0.0218	significant
**AC**	25.06	1	25.06	0.5208	0.4939	
**BC**	0.0048	1	0.0048	0.0001	0.9923	
**A^2^**	1821.83	1	1821.83	37.86	0.0005	significant
**B^2^**	2003.38	1	2003.38	41.64	0.0003	significant
**C^2^**	97.05	1	97.05	2.02	0.1985	
**Residual**	336.81	7	48.12			
**Lack of Fit**	335.51	3	111.84			
**Pure Error**	1.30	4	0.3247			
**Cor Total**	11,791.31	16				
**Std. Dev.**	6.94			R^2^		0.9714
**Mean**	52.54			$R_{aj}^{2}$		0.9347
**C.V (%)**	13.20			**Pred. R^2^**		0.5446
				**Adeq precision**		15.4198

**Table 8:** Criteria constraints and optimal solutions.

Name	Goal	Lower Limit	Upper Limit	Solutions	Desirability
**A: Adsorbent dose**	is in range	10	130	124.115	1
**B: Contact time**	is in range	30	360	249.385	1
**C: Initial concentration**	is in range	10	90	34.6527	1
**adsorption efficiency (%)**	maximize	10.387	76.5418	83.7083	1

**Table 9:** Theoretical amounts of metal ions adsorbed by other natural adsorbents compared with the alginate–clay–carrageenan composite.

Adsorbent	Q_max,Phosphate_ (mg/g)	Reference
MK–chitosan beads	92.05	[63]
Fe/Mn composites (TS-N)	26.00	[64]
Activated carbon prepared from Prosopis juliflora	13.55	[65]
Fe_3_O_4_ @SiO_2_–La composite	27.80	[66]
Biomass	30.21	[67]
Zr/quaternary ammonium powder with polyvinylidene fluoride	15.58	[68]
Magnetite modified with polyacrylamide	28.55	[69]
Magnesium–alginate/chitosan modified biochar microspheres	46.56	[70]
Alginate–carrageenan–clay composite	140.84 (H_2_PO_4_^−^)	This study
	105.26 (HPO_4_^2−^)	

## Data Availability

Data can be provided upon request.

## Supplementary Materials

The following supporting information can be downloaded at: https://www.mdpi.com/article/10.3390/polym17131805/s1, Figure S1: Fourier-transform infrared spectroscopy (FTIR)spectrum of alginate Figure S2: Vanʹt Hoff law for phosphate ion uptake on the engineered bio-composite, and Figure S3 Ramps of optimal solution with desirability = 1.
