# Supplementary material for: Eco-Engineered Biopolymer–Clay Composite for Phosphate IonRemoval: Synergistic Insights from Statistical and AI Modeling
Source: Polymers (Basel). 2025 Jun 28;17(13):1805. doi: 10.3390/polym17131805 (PMC12251723; doi:10.3390/polym17131805)
Supplement: Supplementary file 1 [file polymers-17-01805-s001.zip › polymers-3700921-supplementary.pdf]

Supplementary data

Eco-Engineered Biopolymer–Clay Composite for Phosphate Ions Removal: Synergistic Insights from Statistical and AI Modeling

Rachid Aziam [1,2], Daniela Simina Stefan [1,*], Safa nouaa [2], Mohamed Chiban [2,*] and Mircea Stefan [3]

[1] Department of Analytical Chemistry and Environmental Engineering, Faculty of Chemical Engineering and Biotechnologies, National University of Science and Technology Politehnica of Bucharest, 1–7 Polizu Street, 011061 Bucharest, Romania; rachid.aziam@edu.uiz.ac.ma
[2] Laboratory of Applied Chemistry and Environment, Department of Chemistry, Faculty of Science, Ibnou Zohr University, BP 8106 Agadir, Morocco; safa.nouaa@edu.uiz.ac.ma
[3] Pharmacy Faculty, University Titu Maiorescu, No. 22 Dâmbovnicului Street, District 4, 040441 Bucharest, Romania, mircea.stefan@prof.utm.ro
* Correspondence: daniela.stefan@upb.ro (D.S.S.); m.chiban@uiz.ac.ma (M.C.)

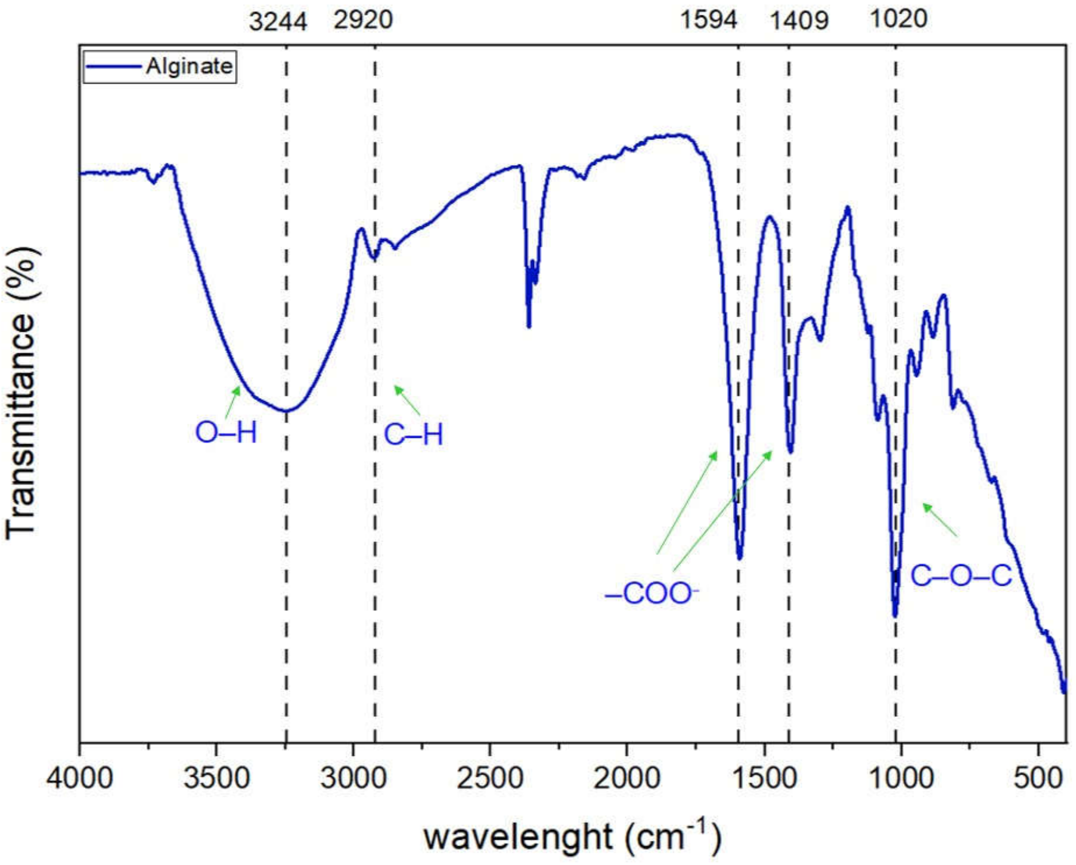

Figure S1. Fourier-transform infrared spectroscopy (FTIR) spectrum of alginate.

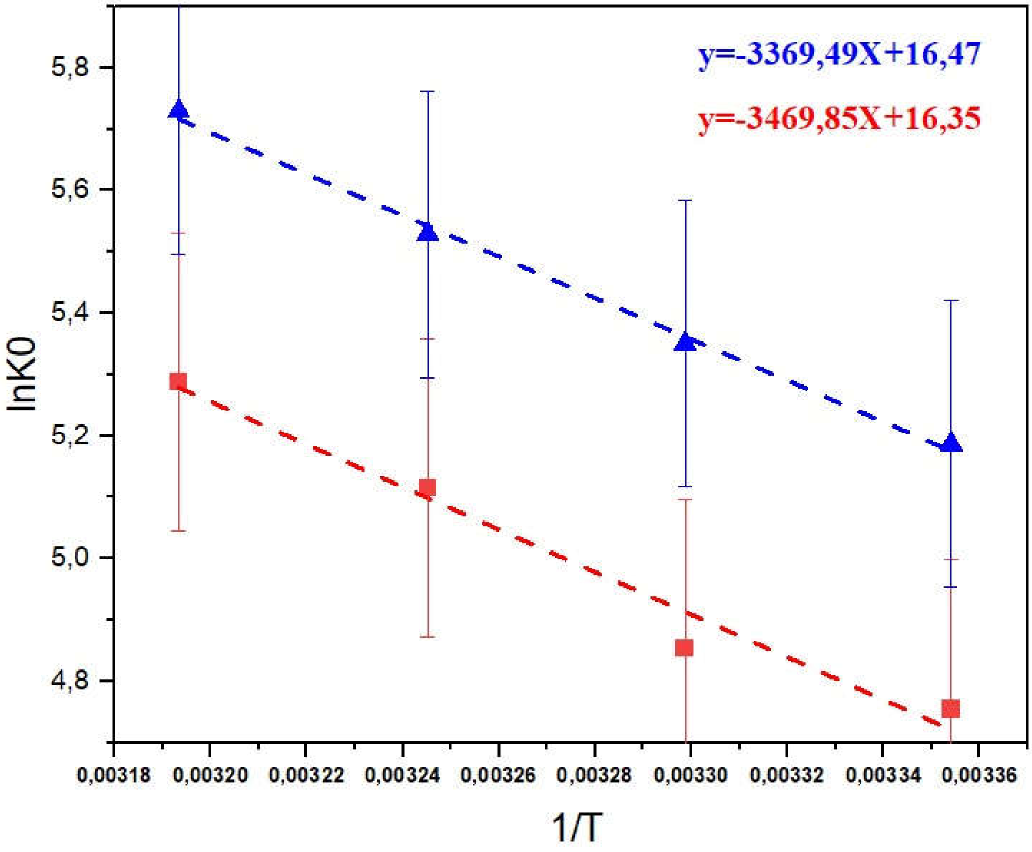

Figure S2: Van't Hoff law for phosphate ions uptake on engineered biocomposite.

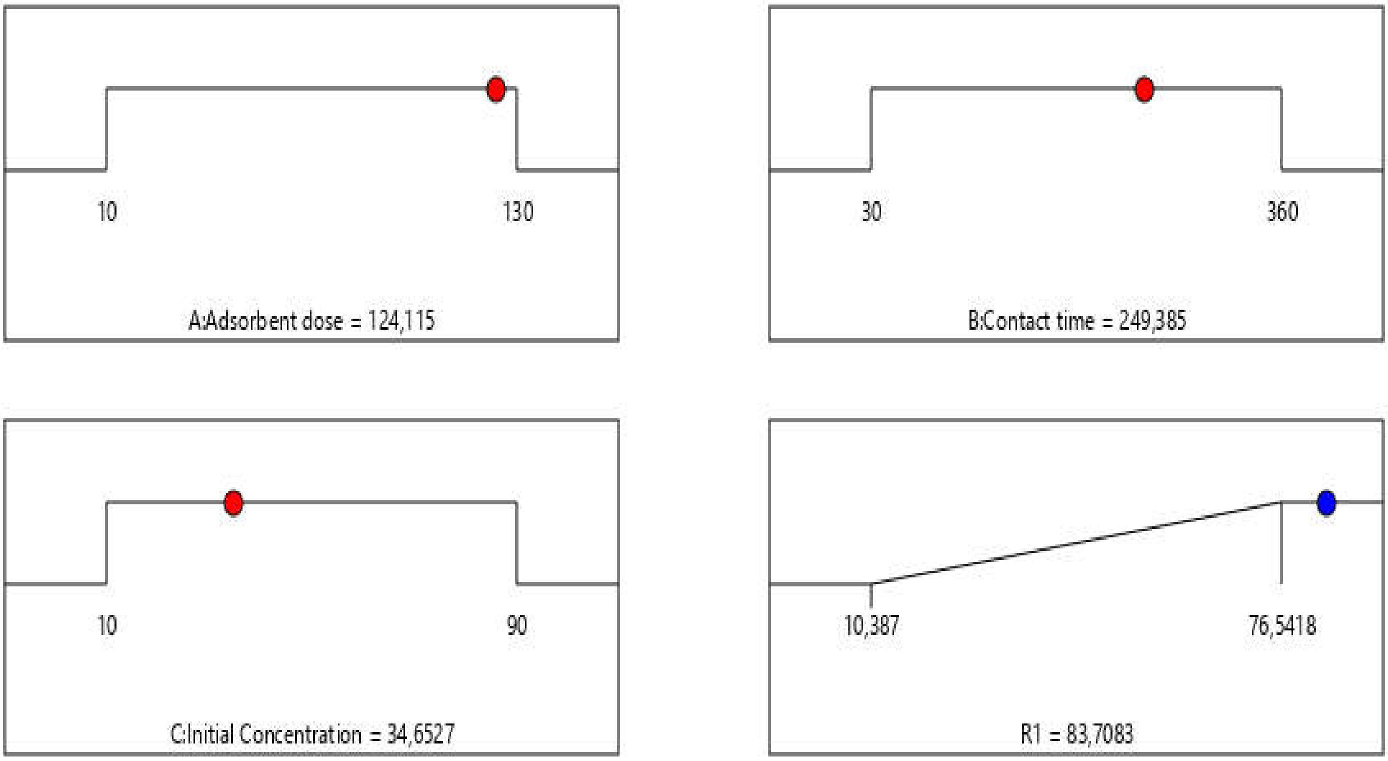

Figure S3. Ramps of optimal solution with desirability = 1.
